# Numerical Laser Energy Deposition on Supersonic Cavity Flow and Sensor Placement Strategies to Control the Flow

**DOI:** 10.1155/2013/141342

**Published:** 2013-12-02

**Authors:** Ibrahim Yilmaz, Selin Aradag

**Affiliations:** Department of Mechanical Engineering, TOBB University of Economics and Technology, Sogutozu Caddesi No. 43, 06560 Ankara, Turkey

## Abstract

In this study, the impact of laser energy deposition on pressure oscillations and relative sound pressure levels (SPL) in an open supersonic cavity flow is investigated. Laser energy with a magnitude of 100 mJ is deposited on the flow just above the cavity leading edge and up to 7 dB of reduction is obtained in the SPL values along the cavity back wall. Additionally, proper orthogonal decomposition (POD) method is applied to the *x*-velocity data obtained as a result of computational fluid dynamics simulations of the flow with laser energy deposition. Laser is numerically modeled using a spherically symmetric temperature distribution. By using the POD results, the effects of laser energy on the flow mechanism are presented. A one-dimensional POD methodology is applied to the surface pressure data to obtain critical locations for the placement of sensors for real time flow control applications.

## 1. Introduction

Cavity configuration is an important configuration for real life applications of aeronautics and it is one of the vital problems of air vehicles which carry stores internally. When air vehicles release the stores from their internal carriages at supersonic speeds, a turbulent, three-dimensional flow occurs over the cavity with problems such as pressure fluctuations and relatively high sound pressure levels. Additionally, the complex flow field occurring in the cavity region decreases the chance of success of the mission of the aircraft. Hence, the control of supersonic cavity flows is crucial.

Many researchers studied supersonic cavity flow to understand the flow mechanism and to develop control strategies. An important study of Aradag [1] includes the cavity configuration with a length to depth (*L*/*D*) ratio of 5.07. The complexity of the cavity flow is presented with CFD results. Ayli [2] performed CFD simulations of cavity configurations with different *L*/*D* ratios. The same cavity configuration used in Aradag's [1] study is also examined in the study of Ayli [2]. Pressure oscillations and sound pressure level (SPL) distributions in specific regions of the cavity are presented.

A complex flow field is observed in the cavity and this leads to pressure oscillations inside the cavity. To suppress these oscillations, various flow control techniques are applied by researchers. Important work related to the cavity control techniques can be found in the study of Yilmaz et al. [3]. Although several techniques are successful for cavities, they may change the flow physics and can result in unexpected effects. Laser energy has been used as an energy deposition method since the discovery of laser induced spark in 1963 [4]. This control technique was studied by several researchers. Adelgren et al. [5] investigated the useful changes in the flow properties of sonic transverse injected wall jet and shock waves in a dual domain interactive space in a supersonic turbulent boundary layer by using laser energy deposition. In another study, to observe the impacts of laser energy deposition on shock waves in supersonic cavity flows, Zaidi et al. [6] performed an experimental analysis. They validated a numerical model with experimental results to be able to use it for future energy deposition studies. An important study is performed by Yan et al. [7]. They deposited the laser pulse to quiescent air and they observed a spherical plasma referring to spherically symmetric temperature profile. They developed a numerical model of laser pulse, which provides a possibility for researchers to study laser energy deposition numerically. Aradag et al. [8] analyzed the effects of laser energy deposition on supersonic cavity flow oscillations. They studied a cavity with *L*/*D* ratio of 5.07 and Mach number of 1.5. 5 to 6 dB reduction is obtained in the sound pressure values. In a similar study, Yilmaz and Aradag [9, 10] applied laser energy deposition method by using the numerical model obtained as a result of the study of Yan et al. [7] to control the pressure oscillations in the cavity region. They also investigated the impacts of frequency, location, and amount of laser energy deposition on an open supersonic cavity flow. At a specific frequency, nearly 3 dB reduction is obtained in the SPL values along the cavity back wall.

The complex cavity flow mechanism and control approaches to suppress the pressure oscillations inside the cavity are broad concerns in the literature. For control approaches especially, mathematical methods are used to obtain reduced order models of the systems. proper orthogonal decomposition method is one of the vital ones. Systems can be represented with fewer data points with the help of POD. Besides being used as a CFD postprocessing tool, by using POD modes and their energy contents, the physics of the systems can be represented. POD technique was first used by Karhunen and Loéve and it was improved by using singular value decomposition and principal component analysis [11–13]. Rowley et al. [14] used POD to obtain reduced order models of different open cavity configurations for flow control approaches. In the study of Nagarajan et al. [15], an open cavity with an *L*/*D* ratio of 2 is modeled by using POD to control the cavity acoustics. Colonius [16] studied active control of open cavities using proper orthogonal decomposition. Kasnakoglu [17] presented several control methods for flow control problems including cavity flow. Yilmaz et al. [18, 19] represented the physics of different cavity flow configurations by using POD. The flow mechanisms inside the cavities are presented using POD modes and energy contents.

This study aims to show the effects of laser energy deposition on an open cavity flow with *L*/*D* ratio of 5.07 and impacts of laser are also shown using POD results. As a continuation of the previous study of Yilmaz and Aradag [9, 10], energy deposition process is examined for longer time periods. The effects of the duration of laser energy on the results are observed. Sensor locations for real time flow control applications are also determined and the results are explained in detail.

## 2. Methodology

**Figure 1:**
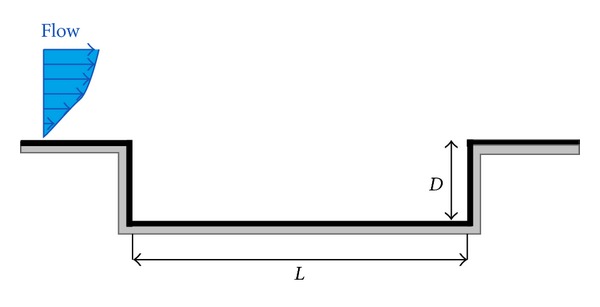
Cavity configuration.

The cavity configuration has an *L*/*D* ratio of 5.07 as shown in Figure 1. The length of the cavity is 0.12065 m and depth is 0.0238 m. The Mach number for the flow is 1.5 and free stream Reynolds number is 1.09 × 10^6^ as summarized by Yilmaz et al. [18, 19].

The CFD simulations of this cavity configuration are performed in the study of Ayli [2]. In the simulations *k*-*ω* turbulence model is used to solve the problem. After laser energy deposition, POD is applied to the *x*-velocity results. POD results of laser energy deposition process are compared with the POD results of the without laser case in the study of Yilmaz et al. [18, 19] to observe the effects of laser energy on the results. A-one dimensional POD methodology is applied to the pressure data which is obtained from the surface of the cavity to specify the critical locations for sensor placement.

### 2.1. Laser Energy Deposition Method

The mathematical model of the laser pulse obtained in the study of Yan et al. [7] is used for the energy deposition process. This model is presented in detail in the previous study of Yilmaz and Aradag [9, 10]. The temperature distribution profile is defined as;

(1) $$\begin{array}{c} \Delta T=\Delta T_{0}e^{-r^{2}/r_{0}^{2}}, \end{array}$$

where *r*_*o*_ is the initial radius and equals 0.45 mm. Δ*T*_0_ is the maximum temperature difference which occurs at given laser location. Δ*T* is the temperature difference and its value depends on the parameter of *r* referring to different locations.

The laser energy is deposited on the cavity 30 times per one Rossiter period. The flow becomes periodic after 12 Rossiter periods as explained in detail in the study of Ayli [2]. The laser energy deposition process is performed along 6 Rossiter periods after the flow becomes periodic.

### 2.2. Proper Orthogonal Decomposition Method (POD)

POD is used for reduced order modeling of the system. This method uses statistical data of the system. The POD method is described in detail in the study of Yilmaz et al. [18, 19]. The characteristics of the flow mechanism are presented with the basis functions *ϕ*_*k*_ and time coefficients *α*_*k*_ obtained as a result of POD. The reconstruction of the systems is made with the following equation [20]:

(2) $$\begin{array}{c} U=\bar{U}+\overset{s}{\underset{k=1}{\sum }}\alpha_{k}\phi_{k},\;k=1,2,\ldots ,s, \end{array}$$

where *U* is the original data set, $\bar{U}$ is the matrix for the mean values, *α*_*k*_ are time coefficients, *ϕ*_*k*_ are basis functions, and *S* is total number of modes.

POD is applied to the *x*-velocity data in the cavity region obtained as results of CFD simulations with laser energy deposition process.

### 2.3. Sensor Placement Methodology

**Figure 2:**
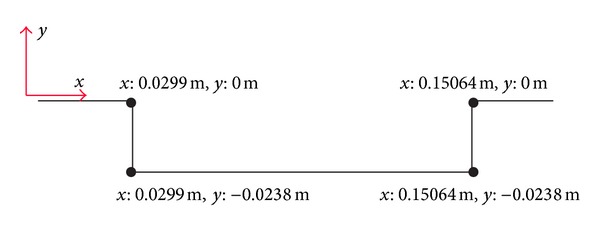
Cavity configuration with coordinates.

For real time control processes, there is a need for efficient data collection. In the cavity configuration, sensors can be installed only on the walls, physically. As in the study of Paksoy [21], which includes the study of the flow around a circular cylinder, sensor locations will be defined with the applications of 1D POD to the pressure values obtained at the cavity leading edge, the cavity floor, and the back wall of the cavity. By examining the POD modes with higher energy contents and defining the locations where these modes are minimum and maximum, the most active locations are determined and sensors are placed at these locations. For the sensor placement process, the geometry of the cavity with coordinates is given in Figure 2.

## 3. Results

### 3.1. Effects of Laser Energy Deposition on the Flow Structure

**Figure 3:**
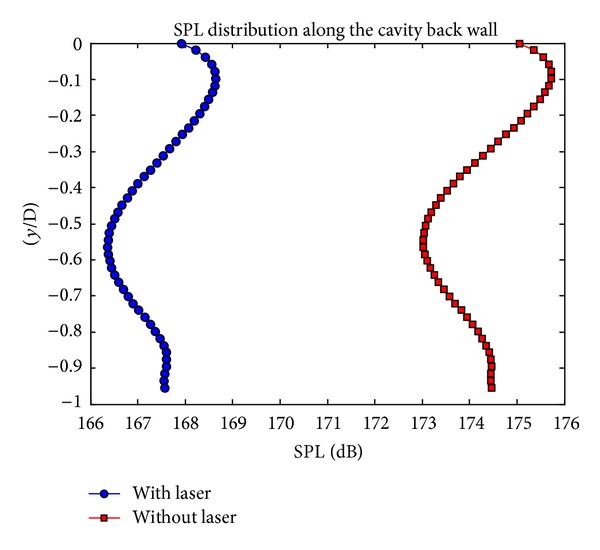
SPL distribution along the cavity back wall.

Energy is deposited to the cavity just above the cavity leading edge as defined in the previous study of Yilmaz and Aradag [9, 10]. Sound pressure level (SPL) values are used as a comparison parameter. SPL distributions with and without laser along the cavity back wall are shown in Figure 3.

Laser energy is deposited at a frequency value of 31110 Hz which means that the laser is deposited to the flow 30 times per period. In the previous study of Yilmaz and Aradag [9, 10], at the same frequency value, nearly 3 dB reduction is obtained. In this study, as it is seen in Figure 3, about 7 dB reduction is obtained in the SPL values. The difference between the results arises from the process time of laser energy deposition. In the present study, laser energy is deposited to the flow along 6 Rossiter periods by increasing the duration of pulsed energy deposition which increases the reduction in SPL values in the pressure oscillations as shown in Figure 3.

**Figure 4:**
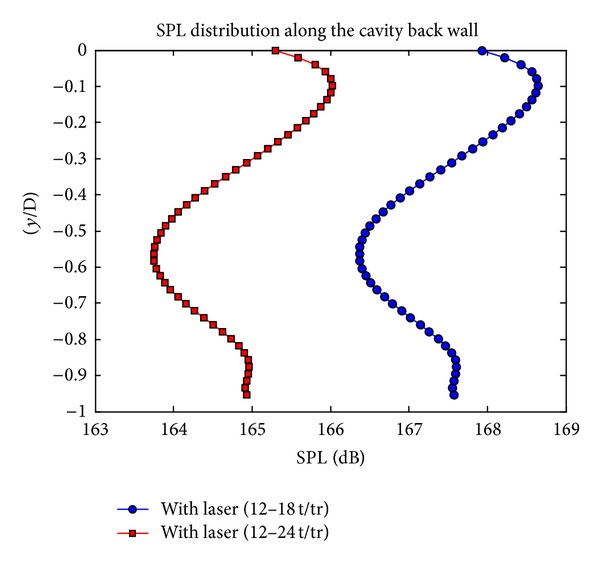
SPL distribution along the cavity back wall (comparison between 12–18 t/tr and 12–24 t/tr).

The laser energy deposition process is performed for six additional periods and as the sound pressure levels are examined, SPL values continue to decrease. In Figure 4, the comparison of the SPL distribution along the cavity back wall, for the process between 12–18 Rossiter periods and 12–24 Rossiter periods, is given.

**Figure 5:**
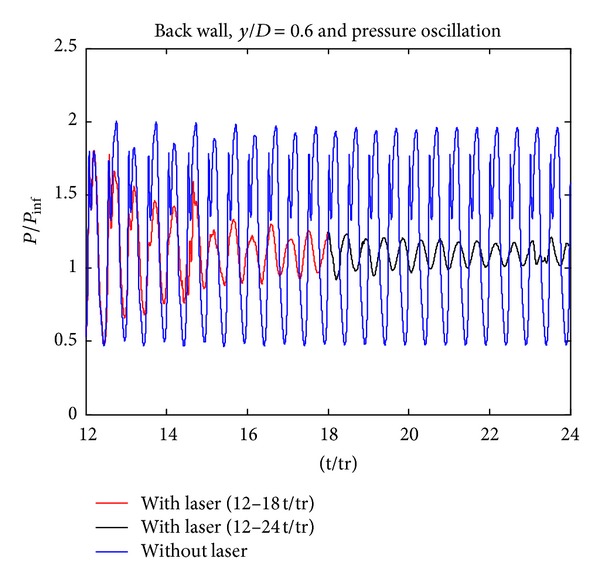
Pressure fluctuations for “with laser” and “without laser” cases (12–24 t/tr).

As it is seen in Figure 4, while continuing the laser deposition process for additional 6 periods, additional 2 dB reduction in SPL values is obtained. In the cavity region, as laser energy is deposited, pressure fluctuations are changed.  Figure 5 shows that the amplitudes of the pressure fluctuations are decreased with laser energy.

### 3.2. POD Results of Cavity Flow with Laser Energy Deposition

**Figure 6:**
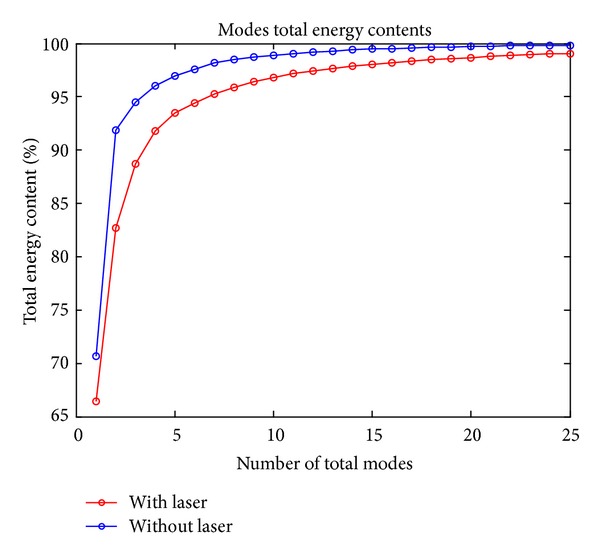
Total energy distribution of POD modes.

The flow mechanism of supersonic cavity flow with *L*/*D* ratio of 5.07 is obtained with CFD simulations. The *x*-velocity data is further examined with the help of a reduced order modeling approach: proper orthogonal decomposition. As a result of POD, energy distribution and energy contents of the modes, each representing different characteristics of the flow, are obtained. The results for the energy distributions of POD modes of cavity flow with laser energy deposition are compared with the results of cavity flow without laser studies that were explained in the study of Yilmaz et al. [18, 19]. The comparison is shown in Figure 6.

As it is seen in Figure 6, energy contents of the modes decrease with the help of laser energy. For the baseline case, without energy deposition, the first mode includes 70.65% of the total energy of the flow while, after laser, the first mode includes 66% of the total energy of the flow. This difference can clearly be seen in Table 1.

**Figure 7:**
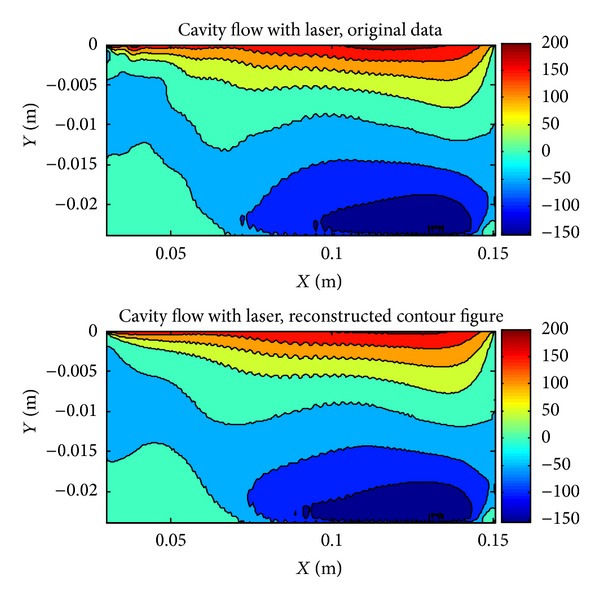
Original *x*-velocity contour and reconstructed *x*-velocity contour using 7 POD modes.

Systems can be represented with modes, the sum of which corresponds to 95-96% of the total energy of the systems, as mentioned in the study of Yilmaz [18, 19]. For the with laser case, comparison of original cavity contour with reconstructed cavity contour using 7 modes is given in Figure 7. There are small differences between reconstructed contour and original contour; therefore the system can be represented with 7 modes which contain 95% of the total energy.

As shown in Table 2, as the total energy content is increased, the number of POD modes of “with laser” case increases faster than “without laser” case.

Tables 1 and 2 show that, to represent the system with laser, more modes are necessary when compared to the baseline case. The energy losses in the first modes and the increase in the necessary number of modes to redefine the system for the case with laser lead to the idea that there is an energy transfer occurring from the dominant structures of the cavity flow to smaller structures. With the laser energy deposition, the flow characteristics are changed and smaller structures become effective on the main characteristics of the flow.

**Figure 8:**
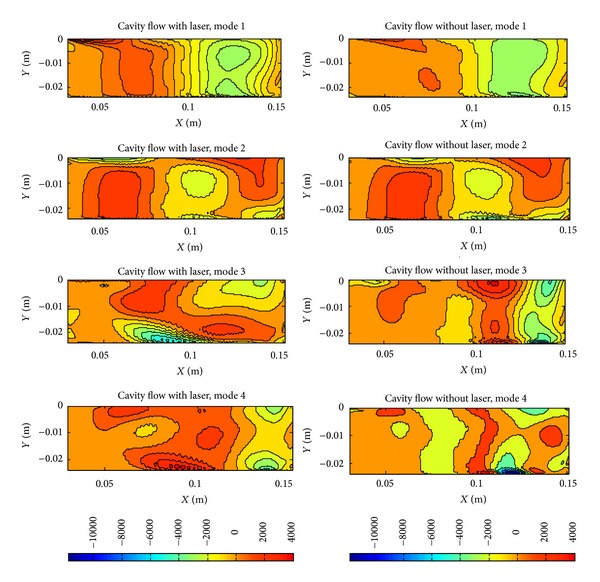
POD modes: with laser and without laser.

The comparison of the first four POD modes is given in Figure 8. While contours of the first two modes include similar structures, other modes show differences.

**Figure 9:**
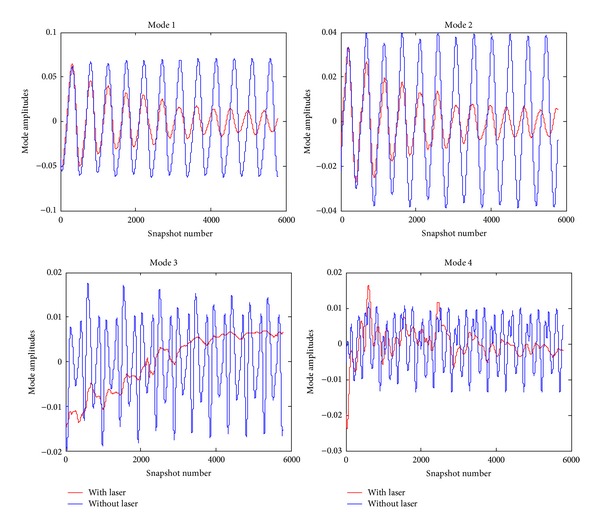
Time coefficient history of POD modes.

By using POD, the structures occurring in the cavity flow can be separated as spatiotemporal. The spatial information is given with the modes in Figure 8. The temporal motions of the modes are presented with data of time coefficient history. In Figure 9, the motions of modes with time are given.

### 3.3. Sensor Placement

After 1D POD is applied to the cavity surface pressure values, the POD modes for “with laser” case and “without laser” case are obtained. The number of POD modes with high energy contents is determined as enough to represent the original data for both cases. To determine the active locations on the surfaces, the minimum and maximum points of POD modes are examined. In Table 3, the POD modes with high energy contents and the coordinates corresponding to the locations where modes are minimum and maximum for “without laser” case are given.

In Table 4, the POD modes with high energy contents and the coordinates, corresponding to the locations where modes are minimum and maximum for “with laser case,” are given.

By examining the location information in Tables 3 and 4, the locations for sensors are determined. Firstly, the most active coordinates which are the same for both cases are chosen. Then, the coordinates which are different, but very close to each other, are specified. The determined sensor locations are presented in Table 5.

**Figure 10:**
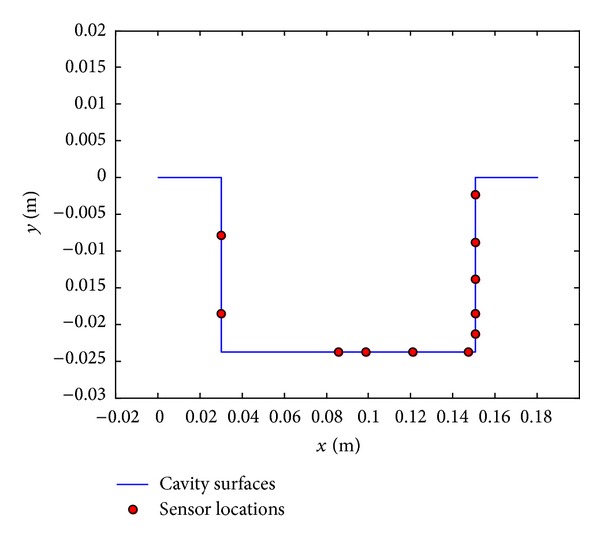
Final sensor placement on the cavity surface.

In Figure 10, final sensor placement on the cavity surface is shown.

## 4. Conclusion

The effects of laser energy on the flow structure are examined for the supersonic flow over an open rectangular cavity. A sound pressure level reduction of 7 dB is obtained at the cavity back wall where pressure oscillations are the highest for the cavity. It is observed that, when the duration of energy deposition is increased, laser is more effective for flow control for cavities. This leads to the idea that the periodicity of the flow may be affected by the laser.

The results of the POD analysis show that, in the case of “with laser,” the necessary number of modes to represent the system increases. While the dominant modes lose impact on the main characteristics of the flow, small structures gain energy and become more effective. The results support the idea that laser energy changes the characteristics of the flow.

Proper orthogonal decomposition is also used to specify the sensor locations. The most active locations on the cavity surfaces are obtained with POD results. The cavity flow mechanism shows that the highest pressure values occur at cavity back wall [2]. So as expected, the critical locations are mainly obtained on the cavity back wall. As a result, sensors are located at these positions.

## Figures and Tables

**Table 1:** Number of modes and energy contents.

Mode number	1	2	3	4	5	6	7	Total energy content, %
With laser, mode energy content, %	66.41	16.25	6.01	3.10	1.68	0.98	0.85	95.28
Without laser, mode energy content, %	70.65	21.19	2.63	1.54	—	—	—	96.01

**Table 2:** Number of modes and energy contents.

	Case of without laser [18, 19]	Case of with laser
Number of modes	12	25
Total energy content, %	99.18%	99.05%

**Table 3:** Number of modes and energy contents with locations corresponding to minimum and maximum points of modes (without laser).

Mode number	Energy contents, %	Coordinates *x* (m)	Coordinates *y* (m)
1	73.88	0.15064	−0.0023
		0.15064	−0.0139
		0.0986	−0.0238
		0.1473	−0.0238
2	19.02	0.15064	−0.0046
		0.0866	−0.0238
		0.119	−0.0238
		0.1478	−0.0238
3	2.24	0.15064	−0.0185
		0.0810	−0.0238
		0.1009	−0.0238
		0.1251	−0.0238
Total, %	**95.14**	—	—

**Table 4:** Number of modes and energy contents with locations corresponding to minimum and maximum points of modes (with laser).

Mode number	Energy contents, %	Coordinates *x* (m)	Coordinates *y* (m)
1	68.34	0.15064	−0.0023
		0.15064	−0.0139
		0.0921	−0.0238
		0.1473	−0.0238
2	16.04	0.15064	−0.0042
		0.15064	−0.0213
		0.0856	−0.0238
		0.1209	−0.0238
3	5.05	0.15064	−0.0088
		0.15064	−0.0199
		0.0861	−0.0238
		0.1227	−0.0238
4	1.7	0.0299	−0.0019
		0.0299	−0.0079
		0.0299	−0.0130
		0.0299	−0.0185
Total, %	**91.13**	—	—

**Table 5:** Number of modes and energy content with locations corresponding to minimum and maximum points of modes.

Coordinates, *x* (m)	Coordinates, *y* (m)	Location
0.15064	−0.0023	Cavity back wall
0.15064	−0.0088	Cavity back wall
0.15064	−0.0139	Cavity back wall
0.15064	−0.0185	Cavity back wall
0.15064	−0.0213	Cavity back wall
0.0856	−0.0238	Cavity floor
0.0986	−0.0238	Cavity floor
0.1209	−0.0238	Cavity floor
0.1473	−0.0238	Cavity floor
0.0299	−0.0079	Cavity leading edge
0.0299	−0.0185	Cavity leading edge
